# Assessment of surface EMG biomarkers in sarcopenic motor dysfunction during postural stabilization

**DOI:** 10.1007/s11357-025-01841-0

**Published:** 2025-08-28

**Authors:** I. Junquera-Godoy, J. L. Martinez-De-Juan, G. González Lorente, I. C. Vendramini, E. M. Scheeren, G. Prats-Boluda

**Affiliations:** 1 Centro de Investigación e Innovación en Bioingeniería, Universitat Politècnica de València, Ci2B) Camino de Vera s/n Ed. 8B, 46022 Valencia, Spain; 2 Graduate Program in Health Technology, Pontifícia Universidade Católica Do Paraná, Curitiba, PR 80215-901 Brazil

**Keywords:** Electromyography, Sarcopenia, Biomarker, Motor control, Postural perturbation

## Abstract

**Purpose:**

This study aimed to investigate neuromuscular adaptations in individuals with pre/sarcopenia during postural balance perturbations, using surface electromyography (sEMG) signal features as potential functional biomarkers of early motor decline.

**Methods:**

Twenty-eight older adults (14 pre/sarcopenic, 14 controls) were subjected to a series of forward balance perturbations while standing on a force platform. sEMG signals were recorded from four lower limb muscles and analyzed across five defined postural epochs established by the perturbation. Six sEMG features were extracted to capture amplitude, frequency, shape, and complexity characteristics of the signals. Linear mixed-effects models were used to evaluate group differences and trial-by-trial adaptation.

**Results:**

The Post-stab epoch (350–2350 ms post-perturbation) revealed the most pronounced differences between groups. The pre/sarcopenic group exhibited significantly lower amplitude and complexity values. Additionally, shape analysis showed a distribution more closely resembling a Laplacian profile in the pre/sarcopenic group, indicative of increased motor unit synchronization and diminished recruitment variability.

**Conclusion:**

This study identifies specific sEMG-derived features, particularly signal shape and complexity metrics, as potential non-invasive biomarkers for neuromuscular decline in sarcopenia. The Post-stab epoch emerges as a sensitive window for detecting deficits in motor control, supporting the use of perturbation-based tasks and sEMG analysis for early detection, monitoring, and intervention planning in aging populations.

**Graphical Abstract:**

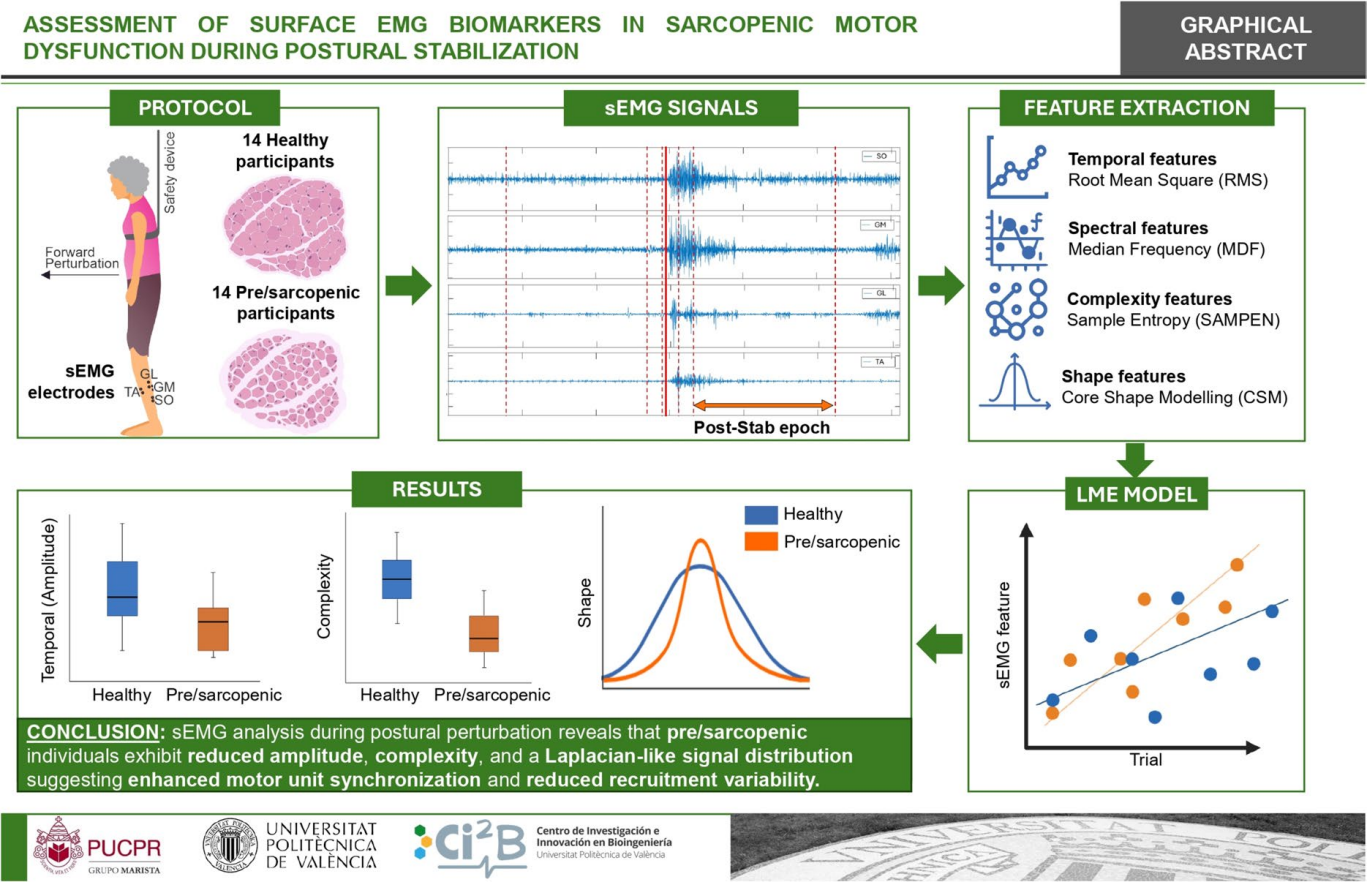

## Introduction

Sarcopenia is a progressive and generalized skeletal muscle disorder characterized by the accelerated loss of muscle mass, strength, and physical performance [1]. While it was initially described as an age-related condition, sarcopenia is now recognized as a complex, multifactorial disorder influenced by chronic diseases, physical inactivity, nutritional deficiencies, and hormonal imbalances [2]. At the cellular level, sarcopenia is driven by a disruption in the balance between muscle protein synthesis and degradation, leading to reductions in the size and number of muscle fibers—particularly type II fibers—combined with increased fat infiltration and altered mitochondrial function [3]. Impairments in neuromuscular signaling and satellite cell activity exacerbate muscle dysfunction and regeneration deficits, underscoring the intricate pathogenesis of the condition [4].

Recent literature increasingly underscores that neuromuscular impairment, not just loss of muscle mass, is a core driver of reduced mobility and functional decline in older adults with sarcopenia. Age-related denervation and deterioration of motor unit function, along with structural and transmission defects at the neuromuscular junction (NMJ), lead to slowed activation rates and diminished muscle power, especially in leg extensors, beyond what can be explained by atrophy alone [5]. In mobility-limited older cohorts, deficits in the rate of neuromuscular activation correlate strongly with reduced gait speed, impaired capacity for chair-rise and stair negotiation, and elevated fall risk, even when single fiber contractile capacity remains relatively preserved [6]. Mechanistic neurophysiological reviews confirm that central motor neuron loss, reduced firing rates, and altered intermuscular coordination disrupt motor output timing and muscle recruitment, thereby undermining functional mobility [7]. Clinically, these findings justify assessing neuromuscular activation (e.g., EMG onset timing or power testing), not only muscle mass, when evaluating sarcopenia because interventions targeting neural as well as muscular components (e.g., resistance training and neuromuscular retraining) yield more robust improvements in gait, balance, and independence [8].

The prevalence of sarcopenia varies widely depending on the diagnostic criteria used. The pooled prevalence across all definitions was estimated at approximately 10% (95% CI 7%–12%) and 16% (95% CI 15%–17%) of the worldwide population [9]. Approximately 5–13% of individuals in their 60 s are affected by sarcopenia, and this prevalence rises to 11–50% in those over 80 years old [10]. Prevalence is notably higher in specific populations, such as hospitalized or institutionalized older adults, where the condition is compounded by comorbidities and reduced mobility [11].

Sarcopenia imposes a significant economic burden on healthcare systems worldwide. In Europe, its financial impact is substantial. For example, the UK incurs approximately £2.5 billion annually in healthcare costs attributable to sarcopenia [12]. In Portugal, sarcopenia increases hospitalization costs by 34–58.5%, depending on the patient’s age [13]. These figures highlight the dual burden of sarcopenia on individuals and society, emphasizing the need for preventive strategies and cost-effective interventions.

One of the most critical health consequences of sarcopenia is the heightened risk of falls [4]. Muscle weakness, combined with diminished balance and coordination, significantly increases the likelihood of falls, which are among the leading causes of injury, hospitalization, and mortality in older adults [14]. Beyond their immediate physical and psychological impact, falls often precipitate a cascade of functional decline, loss of independence, and greater dependency on healthcare resources [14].

Advances in diagnostic techniques have improved the identification and classification of sarcopenia. Grip strength measurement remains a cornerstone for assessing muscle strength, with strong predictive value for adverse outcomes such as disability and mortality [15]. Dual-energy X-ray absorptiometry (DXA) is widely regarded as the gold standard for measuring muscle mass, while bioelectrical impedance analysis (BIA), MRI, and CT scans provide additional diagnostic insights [1]. However, these assessments have several limitations: DXA exposes patients to ionizing radiation, and both DXA and MRI are relatively costly, depend on trained professionals, and are less accessible in routine clinical settings. Moreover, traditional methods often rely on static measurements or anatomical imaging that do not capture the dynamic and functional aspects of neuromuscular performance. For example, dynamometry assesses maximal force in isolated conditions without reflecting muscle coordination during real-world movements. Other emerging methods, including ultrasound and molecular biomarkers, show promise for broader clinical use but require further research and standardization.

In contrast, surface electromyography (sEMG) is a non-invasive, cost-effective technique that records the electrical activity of muscles, providing real-time information about muscle recruitment, coordination, and neuromuscular adaptations [16]. In the context of sarcopenia, sEMG offers unique advantages by detecting impairments in dynamic tasks relevant to mobility and fall risk, allowing for a more comprehensive and functional evaluation of age-related neuromuscular decline.

Several studies have explored the use of sEMG to assess neuromuscular alterations associated with sarcopenia, targeting a variety of muscles, predominantly in the lower limbs due to their role in mobility and postural stability [17–21]. sEMG recordings have been obtained under both isometric and dynamic tasks, including gait, sit-to-stand transitions, and strength evaluations [22–24], with findings often showing reduced muscle activation, altered frequency content, and impaired coordination in sarcopenic individuals [23, 25, 26]. The majority of these studies emphasize the discriminative capacity of classical sEMG features using machine learning classifiers [18, 19, 23, 27, 28], with less attention given to the physiological interpretation of these metrics or their relationship to functional impairments. Very few studies have examined sEMG patterns during dynamic balance perturbation tasks, which are directly relevant to fall risk, or included pre-sarcopenic individuals, thus limiting our understanding of early neuromuscular decline [17, 29]. These gaps support the development of studies that characterize sEMG-derived biomarkers during postural challenges, with the objective of identifying physiological alterations that may precede clinically defined sarcopenia.

To characterize neuromuscular alterations in sarcopenia, a multimodal analysis of sEMG signals offers valuable insight across multiple dimensions. Metrics from the temporal domain, such as root mean square, reflect the level of muscle activation and motor unit recruitment [30]. Spectral features, like median frequency, provide information on muscle fiber conduction velocity and cellular excitability [31]. Complexity measures, such as sample entropy, offer insight into the adaptability and variability of motor control [32]. Finally, shape descriptors, including the central, right, and left shape distance, quantify morphological variability in the sEMG waveform, which relates to motor unit firing patterns and synchronization [33]. This integrated approach allows for a more comprehensive understanding of neuromuscular status.

This study aims to analyze the muscular response of pre/sarcopenic individuals during balance perturbation scenarios and evaluate the progression of motor learning in their leg muscles through a block sequence. By exploring the neuromuscular adaptations associated with balance challenges, the research seeks to provide insights into the mechanisms underlying sarcopenia-related decline and to inform the development of targeted rehabilitation strategies. It is hypothesized that individuals with pre-sarcopenia or sarcopenia will exhibit altered neuromuscular responses and a different motor pattern when compared to healthy controls. The final goal is to enhance functional recovery, reduce fall risk, and improve the quality of life for those affected by sarcopenia.

## Materials and methods

### Participants

The study includes 14 subjects with pre-sarcopenia or sarcopenia and 14 subjects without sarcopenia (Table 1). All participants had no history of neurological disorders, vestibular issues, visual impairments, or dizziness symptoms, and none was using medications that could affect balance. Furthermore, they had not engaged in any physical exercise over the past 6 months.

**Table 1:** Participants of the study

Groups	# Subjects	Sex (M/F)	Age (years)	Body mass (kg)	Height (m)
Pre/sarcopenia	14	3/11	70.21 ± 2.80	75.04 ± 12.15	1.57 ± 0.09
Controls	14	7/7	68.85 ± 3.50	70.90 ± 13.08	1.61 ± 0.07

Participants were recruited from Hospital Nossa Senhora da Luz in Curitiba, Brazil. The study received approval from the Research Ethics Committee (approval no. 4,121,832), and all participants provided written consent before taking part in the study.

Sarcopenia screening followed the guidelines of the European Working Group on Sarcopenia in Older People (EWGSOP) [1]. All participants completed a full battery of assessments: SARC-F questionnaire, handgrip strength, 5 × chair stand test, calf circumference, and the Short Physical Performance Battery (SPPB). Classification was performed after data collection, based on the combination of results across domains. Participants were classified as pre-sarcopenic if they presented low muscle strength only (handgrip < 30 kg for men, < 20 kg for women, or 5 × chair stand test > 15 s), regardless of SARC-F score. Those who presented both low muscle strength and low muscle mass (calf circumference < 31 cm), with or without poor physical performance (SPPB ≤ 8), were classified as sarcopenic. Participants who did not meet criteria for low strength or low muscle mass were classified as non-sarcopenic (controls). The results from these tests can be found in Table 2 [34].

**Table 2:** Results from tests (SARC-F, handgrip strength, chair stand, calf circumference, and SPPB) across each population

Group	SARC-F	Hand grip (Kg)	Chair stand (s)	Calf Circ. (cm)	SPPB
Pre/sarcopenia	4.00 ± 1.71	22.36 ± 6.46	16.09 ± 3.54	33.96 ± 3.72	9.57 ± 1.16
Controls	0.93 ± 0.83	32.18 ± 5.64	12.86 ± 1.93	35.00 ± 2.77	10.29 ± 0.99

### Experimental protocol and data acquisition

**Fig. 1:**
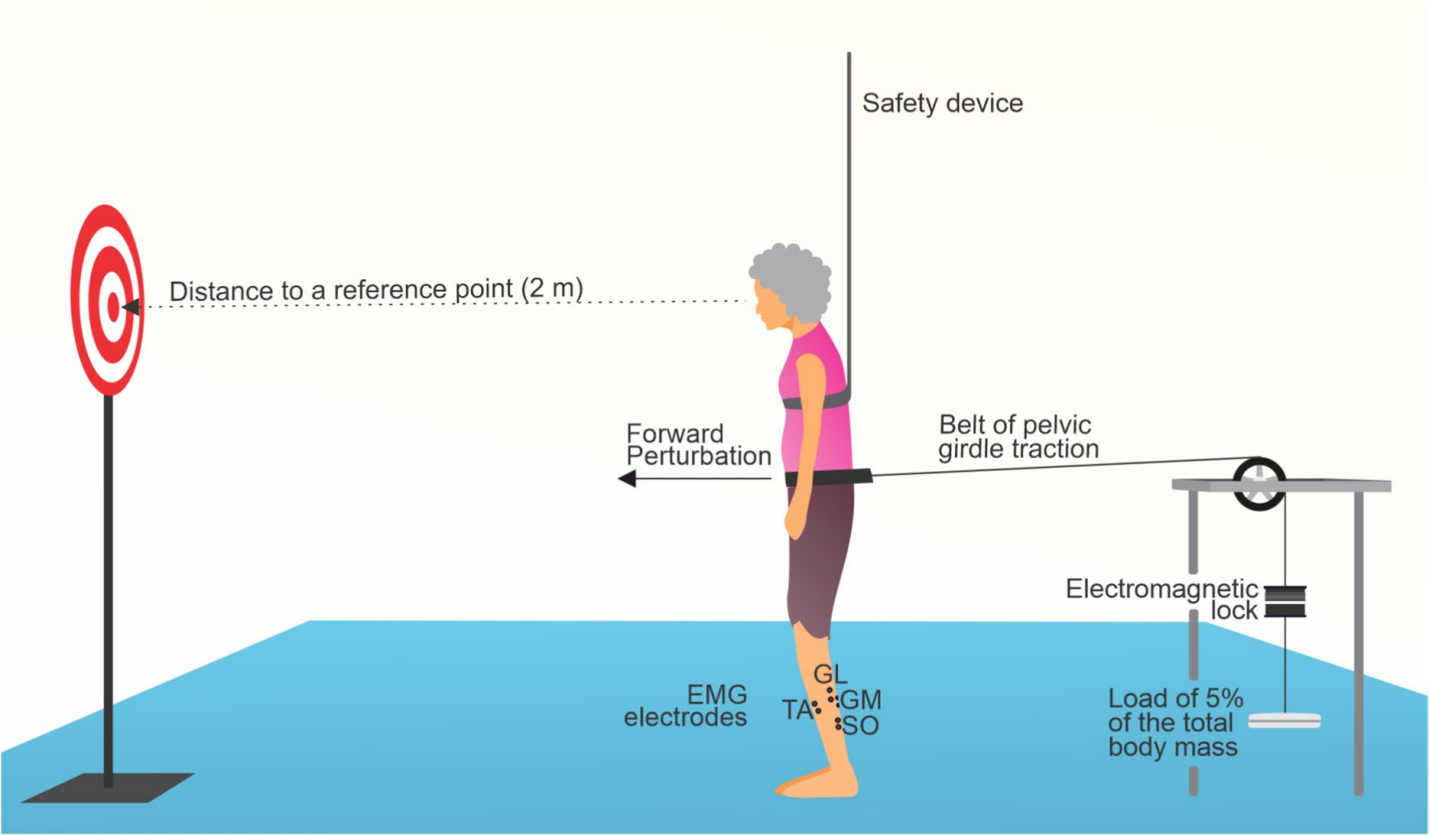
Experimental setup for assessing neuromuscular responses to forward balance perturbations

Surface electromyography (sEMG) signals were recorded using an EMG System do Brasil Ltda. (São José dos Campos, Brazil), model EMG-800C, equipped with passive surface electrodes in a bipolar configuration (Ag/AgCl, 2.2 cm diameter), and placed on the soleus (SO), medial gastrocnemius (GM), lateral gastrocnemius (GL), and tibialis anterior (TA) muscles of the dominant leg. The tibialis anterior and triceps surae muscles were selected based on their key role in the ankle strategy during low-intensity perturbations, the minimal involvement of the knee in such conditions, and their biomechanical alignment with the expected postural responses [35, 36]. The dominant leg was determined through the Waterloo Footedness Questionnaire, as these muscles are essential for ankle movement and balance maintenance in this study. The sEMG signals were amplified by 2000 ×, with an acquisition rate of 2000 Hz. Electrode placement and skin preparation followed SENIAM guidelines, with a reference electrode positioned on the anterior tibia. To introduce balance perturbations, a custom pelvic traction device was used in the Human Motricity Laboratory. Participants stood upright wearing a belt connected to a cable that applied a backward force equal to 5% of their body weight, triggered by a silent electronic device operated by a researcher. This device released the load to initiate the perturbation. Participants positioned their feet on outlined markings, set 17 cm apart and 14° of external hip rotation, and were instructed to focus on a target 2 m away. After setup, they received ten balance perturbations forward, with random intervals between 10 and 30 s to minimize anticipatory responses. A safety system (Fig. 1) was in place to prevent falls. To capture the “first trial reaction,” no warm-up or familiarization was provided prior to data collection, consistent with methods from a previous study [37]. The entire perturbation protocol lasted approximately 3 min. The onset perturbation (defined as time zero) was marked by a synchronization point between the load release trigger and the EMG system.

### Data preprocessing and epoch division

**Fig. 2:**
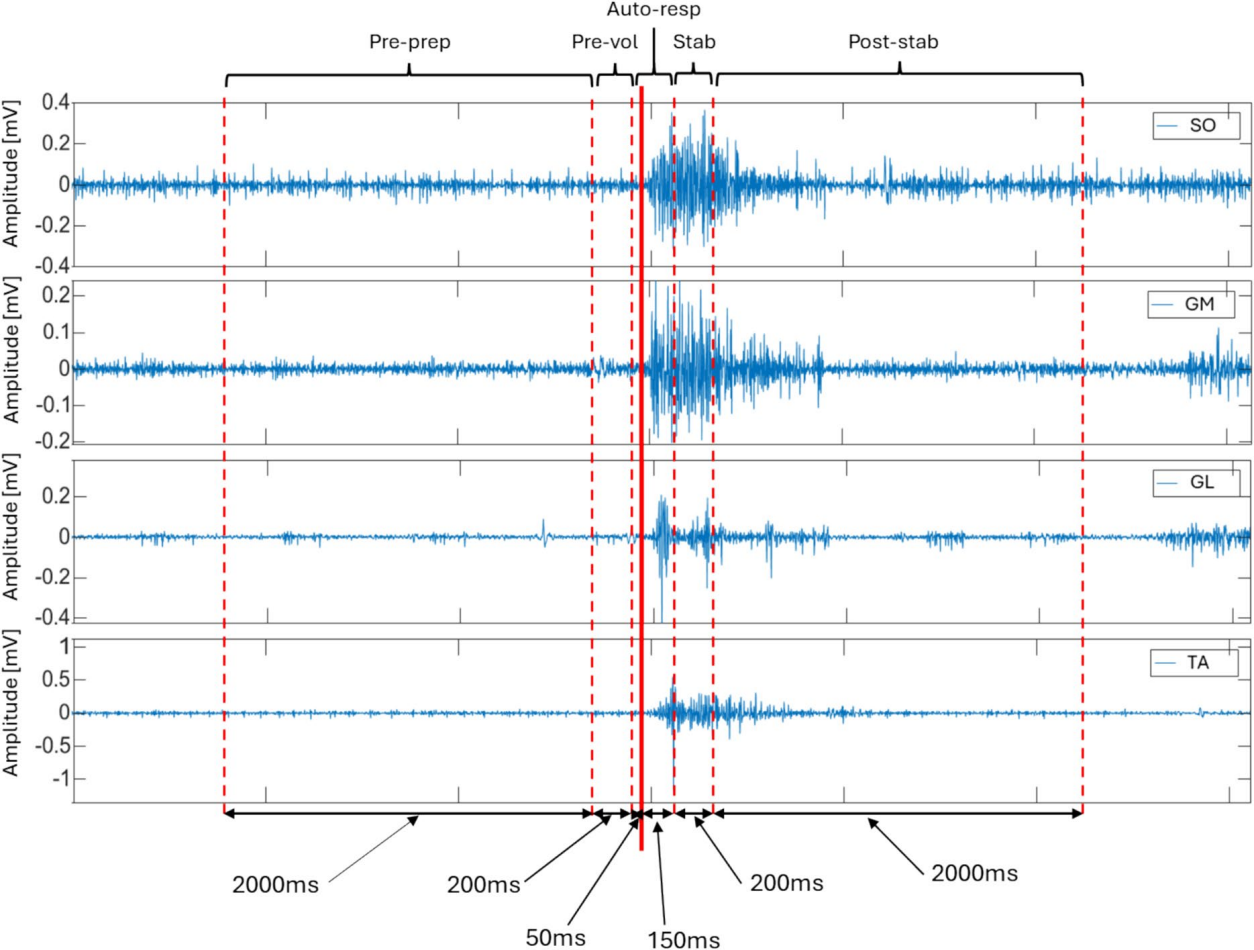
Subdivision of the sEMG signals from the muscles SO, GM, GL, and TA into epochs Pre-prep, Pre-vol, Auto-resp, Stab, and Post-stab from one perturbation

Following data recording, the signals underwent pre-processing to ensure accurate characterization. Initially, a zero-phase Butterworth high-pass filter with a cutoff frequency of 20 Hz was applied, followed by a zero-phase Butterworth low-pass filter at 500 Hz to band-limit the signal. To further eliminate power line interference, a comb filter was used to remove the 60 Hz component and its harmonics. The signals were normalized using a *z*-score and divided into six distinct epochs corresponding to different moments in the protocol as represented in Fig. 2. The characterization of the signals (computation of univariate parameters) was obtained in each of the five epochs.

- Pre-perturbation preparatory epoch (− 2250 ms to − 250 ms)—“Pre-prep”: subtle preparatory adjustments before the perturbation, involving anticipatory motor control to maintain stability.
- Pre-voluntary adjustment epoch (− 250 ms to − 50 ms)—“Pre-vol”: voluntary or pre-voluntary postural adjustments in preparation for the perturbation, optimizing balance control.
- Automatic postural response epoch (− 50 ms to 150 ms)—“Auto-resp”: reflexive postural adjustments triggered by the perturbation, aiming to restore balance through automatic feedback mechanisms.
- Postural stabilization and refinement epoch (150 ms to 350 ms)—“Stab”: refined voluntary postural adjustments to optimize balance, stabilizing the body and correcting posture through feedback mechanisms.
- Post-perturbation stabilization epoch (350 ms to 2350 ms)—“Post-stab”: adaptive postural adjustments for long-term stabilization, involving both feedback and voluntary control to maintain balance.

### Data analysis

#### Temporal features

The amplitude of sEMG signals is determined by various factors, including the number of active motor units, their discharge rates, and the characteristics of the intracellular action potentials (IAPs) [30, 38]. The parameter used to quantify sEMG intensity was the root mean squared value (RMS).

1 $$RMS=\sqrt{\frac{1}{n}\sum_{n}{x}_{n}^{2}}$$

#### Spectral features

Spectral analysis of sEMG signals provides insights into muscle excitability, identifying changes in frequency content and assessing muscle performance. It also aids in studying motor control and coordination by analyzing frequency characteristics during different tasks [31]. The median frequency and spectral moment ratio were computed to analyze the spectral characteristics of the sEMG signals. Median frequency (MDF): frequency at which the total power is divided into two equal halves.

2 $${\int }_{f1}^{MDF}PSD\left(f\right)df= {\int }_{MDF}^{f2}PSD\left(f\right)df$$

where PSD is the power spectral density of the signal and $f1$ and $f2$ define the highest and lowest signal frequencies, respectively.

#### Complexity features

Complexity measures in sEMG quantify the intricacy of muscle activity patterns and assess various aspects of the signal, such as irregularity and randomness, to obtain insights into the complexity of motor unit recruitment and coordination [32]. The sample entropy and Higuchi fractal dimension are computed to analyze the complexity of the signals. Sample entropy (SAMPEN) evaluates the regularity and predictability of muscle activity patterns and quantifies the likelihood of similar signal patterns occurring within a defined tolerance level. Sample entropy analysis in EMG provides valuable information on the stability and variability of muscle contractions and aids in assessing neuromuscular control and identifying abnormalities in motor unit recruitment.

3 $$SampEn=-\mathrm{log}\frac{\sum {A}_{i}}{\sum {B}_{i}}$$

where *A*_i_ is the number of matches of length m + 1 with the *i*^th^ template and *B*_i_ is the number of matches of length m with the *i*^th^ template, *m* is the length of sequences to be compared, and *r* is the tolerance or similarity criterion for data point comparisons. For this, *m* and *r* were set to 1 and 0.1, respectively.

#### Shape features

Recent research has identified potential alterations in the probability density function (PDF) of sEMG data in contexts such as fatigue and muscle force increase [39]. A new method of analyzing PDF shape modifications was proposed using the core shape model (CSM) [33]. The functional statistics used in the present study were the kernel density estimation and the shape distances of probability density functions (PDFs) to assess shape modifications, even for small sample sizes. Three distances were obtained from the CSM method: the central shape distance (CSD), which evaluates PDF peakedness, the left shape distance (LSD), and the right shape distance (RSD), both of which evaluate PDF asymmetry.

4 $${CSD}_{(p,q)}=\sqrt{{\int }_{0.4}^{0.6}{(\alpha {P}^{-1}\left(y\right)+\beta -{G}^{-1}\left(y\right))}^{2}dy}$$

5 $${LSD}_{\left(p,q\right)}=\sqrt{{\int }_{0}^{0.25}{\left(\alpha {P}^{-1}\left(y\right)+\beta -{G}^{-1}\left(y\right)\right)}^{2}dy}$$

6 $${RSD}_{(p,q)}=\sqrt{{\int }_{0.75}^{1}{(\alpha {P}^{-1}\left(y\right)+\beta -{G}^{-1}\left(y\right))}^{2}dy}$$

where *P* and *G* are the PDF of the sEMG signal and a Gaussian PDF, respectively. In the continuous domain, these PDFs are defined as *p* and *q*. The *α* and *β* values are approximated using a constrained linear regression between ${P}^{-1}(\mathrm{x})$ and ${G}^{-1}(\mathrm{x})$.

#### Linear mixed-effects models

In this study, we employed linear mixed-effects models (LMEMs) to analyze the evolution of surface electromyography (sEMG) parameters in two distinct population groups: controls and pre/sarcopenic subjects [40]. Our primary objective was to model the differences in muscle reaction and adaptation across these groups in response to unbalanced conditions, ultimately evaluating each group’s risk of falls. LMEMs were selected for their ability to handle the hierarchical structure of our data, with repeated measures over multiple trials and individual variability among participants.

In this study, we explored the learning capacity of the muscles in response to repeated trials of unbalanced conditions, hypothesizing that muscles in both control and pre/sarcopenic groups would adapt differently. Muscle learning capacity, or motor adaptation, reflects the ability of muscles to adjust their response patterns over time, thereby enhancing stability and reducing fall risk. By examining changes in sEMG parameters over consecutive trials, we aimed to determine if pre/sarcopenic individuals exhibit compromised learning capacity, potentially leaving them at a higher risk of falls compared to the control group. The linear mixed effects model (LMEM) applied in this analysis was specified as:

$$sEMG\_parameter\sim Group(Sarc/Cont)*Trial+(1|Subject)$$

In this model:

- Fixed effects:
  o Group (control/sarcopenic): The group variable captures the overall differences in muscle response between the control and pre/sarcopenic groups.
  o Trial: This variable represents the sequence of trials, allowing us to assess changes in muscle activation over repeated exposures to unbalanced conditions.
  o Group*Trial interaction: The interaction between group and trial enables us to investigate whether the pattern of muscle adaptation (learning capacity) differs between control and pre/sarcopenic individuals over the course of the trials. A significant interaction would indicate that one group may exhibit a steeper or flatter learning curve, reflecting either enhanced or reduced adaptability in response to postural instability.
- Random effects:
  o (1|Subject): The random intercept for each subject accounts for individual differences in baseline sEMG levels, recognizing that each participant may have a unique starting point or overall muscle activation level. This helps to isolate within-subject variation over trials from between-subject variation, providing a clearer view of the learning dynamics across trials.

The inclusion of the Group*Trial interaction term is particularly valuable for examining learning capacity. This model allows us to quantify learning differences in muscle response and provides insights into the capacity of each group to adapt their muscle behavior under challenging conditions.

In this study, we utilized the “*mixedlm*” function from the “*statsmodels*” library in Python to perform our linear mixed effects modelling. This function is specifically designed to handle mixed effects models, allowing us to incorporate both fixed effects, such as group and trial conditions, and random effects to account for individual variability.

### Statistical analysis

For the statistical testing of fixed effects within our model, “*mixedlm*” applies the Wald *z*-test [41]. This test evaluates the significance of each fixed effect by comparing the estimated coefficient to its standard error, generating a *z*-score. The *z*-score is then used to calculate a *p*-value, which indicates whether the effect of each predictor is statistically significant. The Wald *z*-test, as implemented in “*statsmodels*”, assumes an asymptotic normal distribution of the *z*-statistic, making it a suitable choice for our analysis, particularly given the hierarchical structure of our data and the need to test the effects across repeated measures. In linear mixed-effects models (LMEMs), statistical analysis often involves testing the significance of fixed effects, random effects, and interactions to determine the contribution of each predictor to the outcome variable. The *p*-value is a key statistic in this context, as it indicates whether the observed effects are likely to have occurred by chance.

- A significant *p*-value (*p*-value < 0.05) for a fixed effect suggests that the predictor has a meaningful effect on the outcome.
- A significant interaction effect (*p*-value < 0.05) implies that the effect of one predictor on the outcome varies depending on the level of another predictor.

Prior to model generation, potential confounding effects of age and body mass index (BMI) on the sEMG parameters were assessed to ensure that subsequent analyses reflected genuine physiological differences rather than demographic biases. To achieve this, Spearman’s rank correlation was computed between each sEMG variable and the cofactors (age and BMI) [42]. The results of this analysis can be found in Table 6 in the Annex section. In cases where a statistically significant correlation was found (*p*-value < 0.05), the corresponding sEMG variable was adjusted using a generalized additive model (GAM), applying the correction:${Y}_{C}=Y-f(X)$, where $f(X)$ represents a smooth function fitted to the data. Finally, to assess potential sex-related differences in sEMG parameters, a Wilcoxon rank-sum test was conducted. The results of this analysis are presented in Table 7 of the Annex section.

## Results

### Linear mixed effects models

Table 3 displays the *p*-values from the statistical analysis of the linear mixed-effects model (LMEM) for the interaction effect “GroupTrial,” which reflects differences in the evolution of each parameter across trials when comparing the pre-sarcopenic and control groups. The analysis includes six parameters (RMS, MDF, SAMPEN, CSD, RSD, and LSD) across four muscles (TA, GL, GM, and SO) and five inspection epochs (Pre-prep, Pre-vol, Auto-resp, Stab, and Post-stab). Significant *p*-values were most frequently observed for the SAMPEN and CSD parameters across multiple muscles and epochs. For the TA muscle, significant group*trial interactions were identified in the Pre-prep epoch for SAMPEN, CSD, and LSD; in the Auto-resp epoch for RSD; and in the Post-stab epoch for MDF. In the GL muscle, significant interactions were found for CSD in the Pre-prep epoch, SAMPEN in the Pre-vol epoch, and LSD in the Post-stab epoch. The GM muscle exhibited significant interactions in the Pre-vol epoch for CSD and RSD. For the SO muscle, SAMPEN and CSD showed significant differences in the Auto-resp epoch, MDF was significant in the Stab epoch, and SAMPEN was again significant in the Post-stab epoch. Overall, SAMPEN demonstrated the most frequent significant differences, followed by CSD, with key group*trial interactions observed in the Pre-prep, Pre-vol, and Post-stab epochs, particularly in the TA, GL, and SO muscles. These results suggest distinct differences in the evolution of these parameters between the pre-sarcopenic and control groups across trials.

**Table 3:** *P*-values from the statistical analysis of the LMEM of the interaction effect “Group*Trial.” The table is divided into four muscles, the five epochs of inspection, and the six parameters computed. Statistically significant *p*-values (*p* < 0.05) in bold with (*)

Muscle	Epoch	RMS	MDF	SAMPEN	CSD	RSD	LSD
**TA**	**Pre-prep**	0.723	0.500	0.040*	0.049*	0.454	0.010*
	**Pre-vol**	0.613	0.829	0.648	0.199	0.605	0.374
	**Auto-resp**	0.496	0.840	0.428	0.924	0.050*	0.253
	**Stab**	0.465	0.677	0.479	0.768	0.566	0.787
	**Post-stab**	0.507	0.038*	0.379	0.541	0.380	0.104
**GL**	**Pre-prep**	0.834	0.273	0.461	0.045*	0.600	0.891
	**Pre-vol**	0.888	0.091	0.025*	0.716	0.601	0.325
	**Auto-resp**	0.668	0.127	0.985	0.924	0.467	0.784
	**Stab**	0.933	0.294	0.438	0.105	0.439	0.381
	**Post-stab**	0.340	0.353	0.196	0.389	0.813	0.020*
**GM**	**Pre-prep**	0.250	0.912	0.819	0.471	0.973	0.907
	**Pre-vol**	0.946	0.792	0.245	0.004*	0.048*	0.828
	**Auto-resp**	0.977	0.306	0.966	0.203	0.389	0.227
	**Stab**	0.551	0.736	0.295	0.963	0.835	0.631
	**Post-stab**	0.922	0.694	0.187	0.359	0.690	0.814
**SO**	**Pre-prep**	0.672	0.240	0.779	0.378	0.779	0.185
	**Pre-vol**	0.375	0.151	0.087	0.299	0.937	0.276
	**Auto-resp**	0.162	1.000	0.028*	0.032*	0.346	0.154
	**Stab**	0.513	0.032*	0.266	0.884	0.595	0.601
	**Post-stab**	0.471	0.332	0.026*	0.234	0.089	0.159

Table 4 shows the *p*-values from the statistical analysis of the linear mixed-effects model (LMEM) for the fixed effect “Group,” which reflects significant differences in the parameter values between the pre-sarcopenic and control groups. The analysis includes six parameters (RMS, MDF, SAMPEN, CSD, RSD, and LSD) across four muscles (TA, GL, GM, and SO) and five inspection epochs (Pre-prep, Pre-vol, Auto-resp, Stab, and Post-stab). Significant *p*-values were more frequent in the Post-stab epoch across muscles and parameters, with SAMPEN, CSD, RSD, and LSD showing the most differences between groups. For the TA, significant differences in parameter values were observed for CSD in the Pre-prep and Post-stab epochs, and for RMS in the Stab and Post-stab epochs. The GL showed significant group effects in MDF during the Auto-resp and Stab epochs, in SAMPEN during the Post-stab epoch, and in LSD during the Stab and Post-stab epochs.

**Table 4:** *P*-values from the statistical analysis of the LMEM of the fixed effect “Group.” The table is divided into four muscles, the five epochs of inspection, and the six parameters computed. Statistically significant *p*-values (*p* < 0.05) in bold with (*)

Muscle	Epoch	RMS	MDF	SAMPEN	CSD	RSD	LSD
**TA**	**Pre-prep**	0.589	0.836	0.653	0.033*	0.566	0.447
	**Pre-vol**	0.759	0.723	0.362	0.050*	0.919	0.114
	**Auto-resp**	0.396	0.339	0.621	0.195	0.098	0.933
	**Stab**	0.024*	0.654	0.509	0.072	0.283	0.720
	**Post-stab**	0.050*	0.446	0.672	0.017*	0.206	0.184
**GL**	**Pre-prep**	0.782	0.273	0.266	0.444	0.271	0.746
	**Pre-vol**	0.212	0.890	0.173	0.519	0.127	0.497
	**Auto-resp**	0.661	0.020*	0.160	0.519	0.983	0.924
	**Stab**	0.198	0.012*	0.855	0.163	0.251	0.014*
	**Post-stab**	0.612	0.565	0.049*	0.194	0.007*	0.728
**GM**	**Pre-prep**	0.005*	0.532	0.077	0.113	0.598	0.458
	**Pre-vol**	0.147	0.587	0.022*	0.045*	0.002*	0.544
	**Auto-resp**	0.524	0.767	0.065	0.842	0.464	0.722
	**Stab**	0.295	0.907	0.655	0.800	0.719	0.355
	**Post-stab**	**0.037**	0.979	0.002*	0.005*	0.002*	0.233
**SO**	**Pre-prep**	0.133	0.888	0.517	0.022*	0.257	0.093
	**Pre-vol**	0.026*	0.986	0.084	0.760	0.900	0.077
	**Auto-resp**	0.105	0.468	0.085	0.459	0.078	0.887
	**Stab**	0.831	0.039*	0.153	0.049*	0.320	0.299
	**Post-stab**	0.000*	0.923	0.000*	0.015*	0.000*	0.003*

For the GM, significant group differences were found in RMS during the Pre-prep and Post-stab epochs, in SAMPEN during the Pre-vol and Post-stab epochs, and in CSD, RSD, and LSD during the Post-stab epoch. The SO exhibited significant differences in RMS during the Pre-vol and Post-stab epochs, in SAMPEN during the Post-stab epoch, and in CSD, RSD, and LSD also during the Post-stab epoch.

Overall, the Post-stab epoch exhibited the highest frequency of significant *p*-values across parameters and muscles. Parameters such as RMS, SAMPEN, CSD, and RSD consistently demonstrated significant group effects, particularly in the GM and SO muscles. These results highlight that the differences in parameter values between pre-sarcopenic and control groups are most pronounced in later trials.

Based on the count and distribution of significant *p*-values observed in Tables 3 and 4, as summarized in Table 5, the Post-stab epoch was selected for further analysis, as it showed the highest concentration of statistically significant differences between the pre-sarcopenic and control groups. This epoch showed the greatest number of significant Group*Trial interactions (Table 2) and fixed group effects (Table 3) across multiple parameters—particularly SAMPEN, CSD, RSD, and RMS—and across several muscles, most notably the GM and SO.

**Table 5:** Summary of the number of statistically significant effects by epoch

EPOCHS	Group*Trial	Group	Total
Pre-prep	4	3	7
Pre-vol	3	5	8
Auto-resp	3	1	4
Stab	1	5	6
Post-stab	**3**	**13**	**16**

**Fig. 3:**
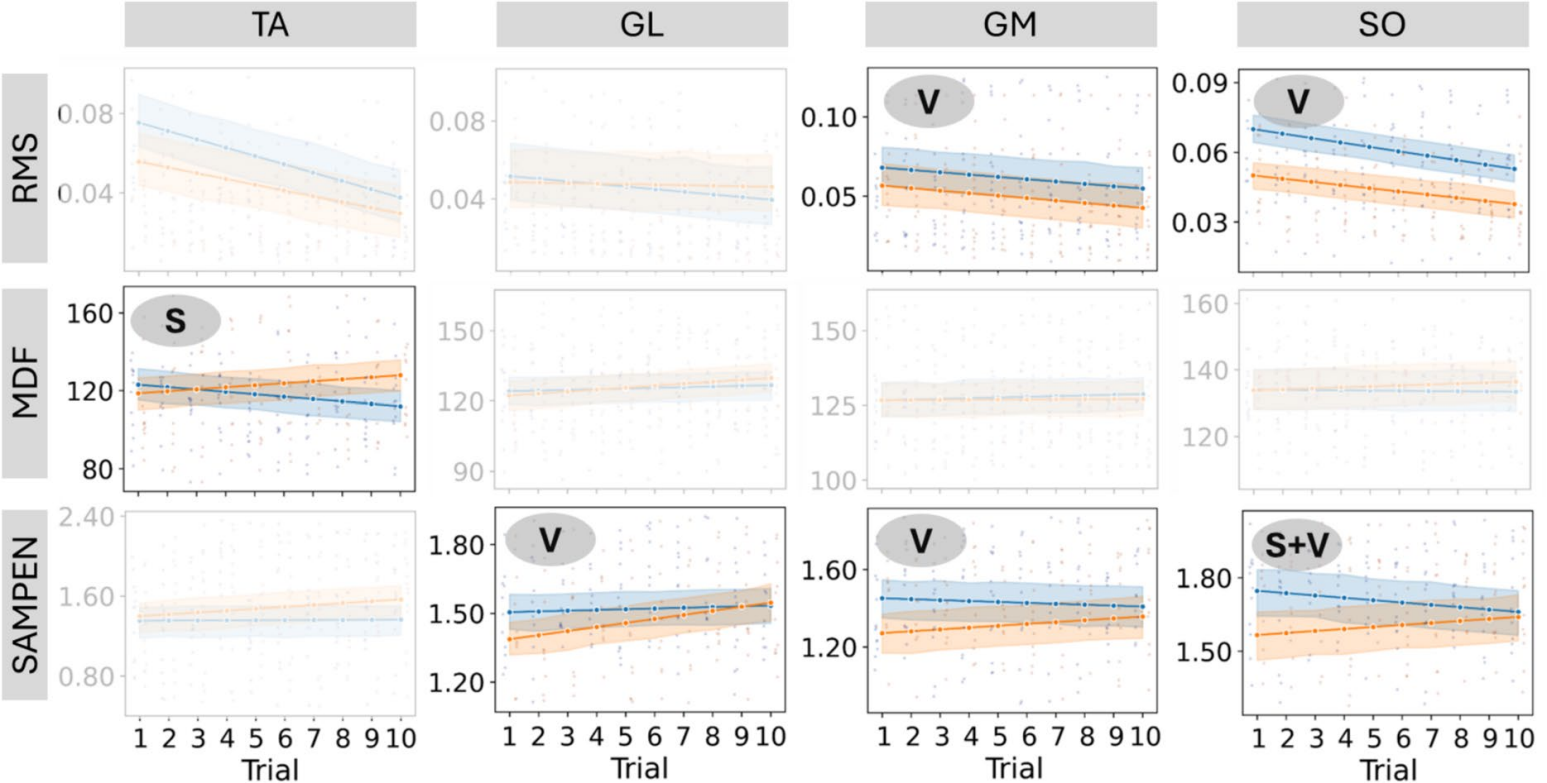
Trends for the pre-sarcopenic (orange) and control (blue) groups during the Post-stab epoch. Columns correspond to the four muscles: TA, GL, GM, and SO; rows correspond to the parameters RMS, MDF, and SAMPEN. Only combinations of muscle and parameter showing at least one significant difference between groups are displayed. Legend: “V” indicates a significant difference in values between groups, “S” indicates a significant difference in slopes, and “V + S” indicates significant differences in both value and slope

Figure 3 gives the results comparing trends and values in three parameters—RMS, MDF, and SAMPEN—across four muscles (TA, GL, GM, and SO) between two groups: pre/sarcopenic (orange) and control (blue) in the Post-stab epoch. Statistically significant differences are highlighted with symbols: “V” indicates a significant difference in the parameter values between the groups. “S” indicates a significant difference in the slope of the trends between the groups. “V + S” indicates significant differences in both the values and the slopes. In the Post-stab epoch, significant group differences were observed across several muscles and sEMG-derived parameters. For the RMS parameter, the GM and SO muscles showed significantly lower values in the pre/sarcopenic group compared to controls. Regarding MDF, only the TA muscle exhibited a significant difference in slope between groups, with a slightly increasing trend observed in pre/sarcopenic individuals. For SAMPEN, significant differences in values were found in the GL, GM, and SO muscles, with the latter also showing a significant difference in slope. Overall, SAMPEN tended to increase across trials in the pre/sarcopenic group, with higher values consistently observed in the control group.

**Fig. 4:**
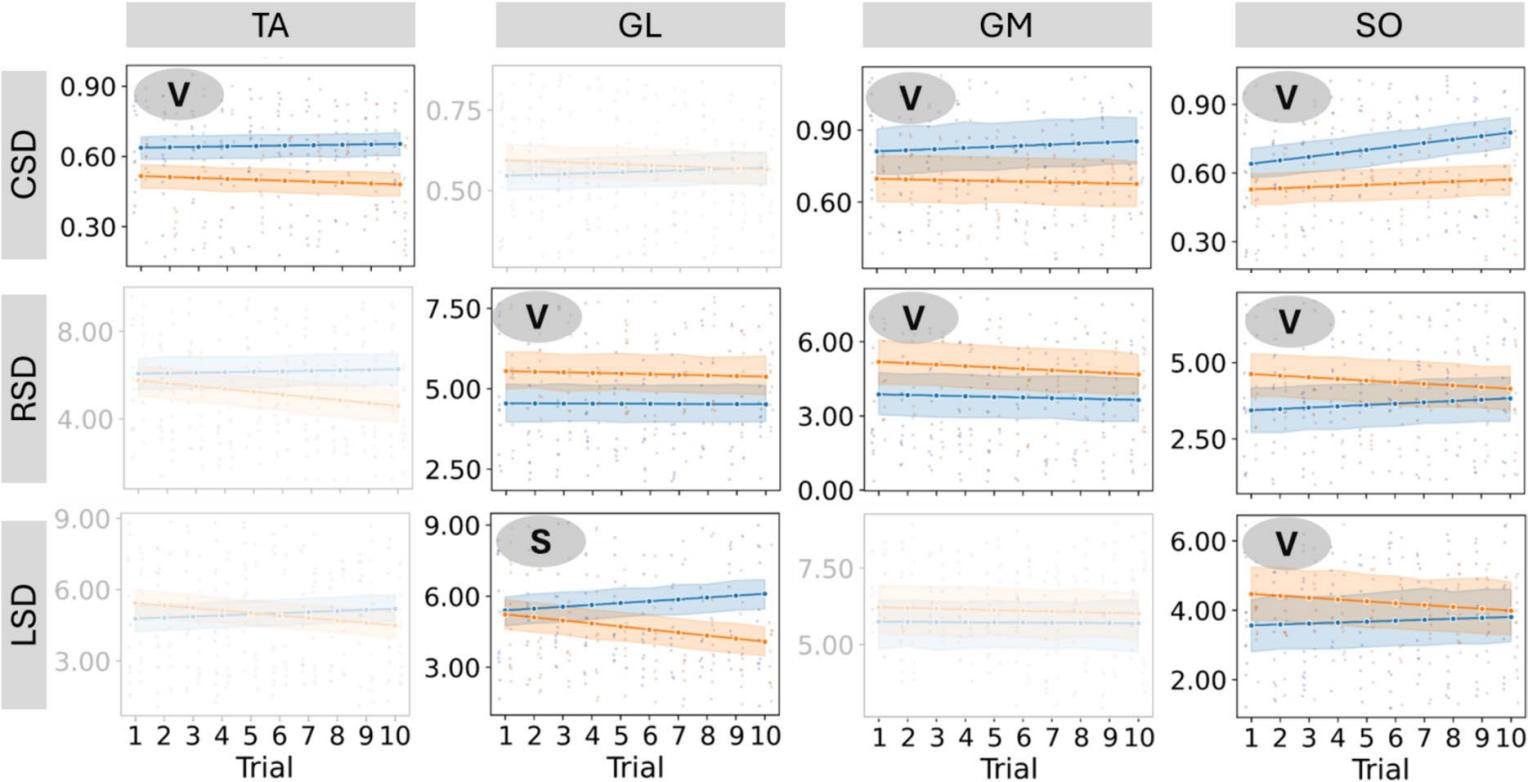
Trends for the pre-sarcopenic (orange) and control (blue) groups during the Post-stab epoch. Columns correspond to the four muscles: TA, GL, GM, and SO; rows correspond to the parameters CSD, RSD, and LSD. Only muscle–parameter combinations with at least one significant difference between groups are shown. Legend: “V” indicates a significant difference in values between groups; “S” indicates a significant difference in slopes

Figure 4 gives the results comparing trends and values in three parameters—CSD, RSD, and LSD—across four muscles (TA, GL, GM, and SO) between two groups: pre/sarcopenic (orange) and control (blue) in the Post-stab epoch. Statistically significant differences are highlighted with symbols: “V” indicates a significant difference in the parameter values between the groups. “S” indicates a significant difference in the slope of the trends between the groups. In the Post-stab epoch, several significant differences in the shape parameters of the sEMG signal distributions were found between pre/sarcopenic and control groups. For the CSD, significantly higher values were observed in the control group for TA, GM, and SO muscles, indicating more pronounced central shape differences. In the case of RSD, significant value differences were found in the GL, GM, and SO muscles, with controls presenting lower RSD values. Lastly, for LSD, significant group differences were detected in the SO muscle, with controls again showing lower values. Additionally, a significant slope difference was found in GL for LSD, where the control group showed an increasing trend, while the pre/sarcopenic group exhibited a decreasing trajectory.

## Discussion

This study aimed to evaluate neuromuscular adaptations in pre-sarcopenic and sarcopenic individuals during balance perturbations by analyzing sEMG signal characteristics. These results from the sEMG analysis expand upon the findings previously reported by Vendramini et al. [34], which focused on postural balance parameters obtained from the same experimental protocol [34]. While that study highlighted biomechanical alterations in balance control, the present work complements it by identifying neuromuscular signal features that may serve as early biomarkers of sarcopenia, with potential applicability in clinical practice.

These results reveal distinct neuromuscular patterns between control and pre/sarcopenic individuals during repeated postural stabilization trials. The lower RMS values in the GM and SO muscles of the pre/sarcopenic group during the Post-stab phase suggest reduced muscle activation, possibly due to decreased motor unit recruitment. Similar findings have been reported in [25], while [27] showed contrasting results. Although both studies examined upper limb muscles, the discrepancy may stem from the differing protocols: [25] assessed a functional upper limb movement, closely aligning with our dynamic lower extremity task, whereas [27] utilized an isometric handgrip contraction. The more functional nature of [25] likely accounts for the alignment in findings despite targeting different extremities.

In contrast, [20] examined lower leg muscles during quiet standing and found increased muscle activity in sarcopenic individuals, likely as a compensatory mechanism to maintain balance. However, their study used a different protocol (no perturbation) and calculated integrated EMG (iEMG) instead of RMS, which may explain the differing outcomes. The findings of [20], which reported increased iEMG during postural tasks in sarcopenic individuals, may indicate a pattern of chronic overactivation or prolonged low-intensity co-contraction aimed at stabilizing posture. In contrast, the decreased RMS observed in our study during balance perturbation suggests an impaired ability to generate adequate phasic muscle activation in response to sudden, high-demand challenges. This may be attributed to delayed motor unit recruitment or diminished neuromuscular reserve, both of which are characteristic of sarcopenia. Importantly, these observations are not necessarily contradictory but may instead reflect different aspects of neuromuscular adaptation: while sarcopenic muscles may compensate during low-intensity, sustained tasks by increasing coactivation, they may concurrently lack the capacity for effective rapid activation when faced with more dynamic or reactive demands.

Similarly, [26] reported a decrease in RMS in lower leg muscles, aligning with the results of our study. Their protocol involved a 30-s continuous sit-to-stand task, which, like our perturbation paradigm, imposed a sustained functional demand on the lower extremities, likely revealing comparable deficits in phasic muscle activation among sarcopenic individuals.

These findings highlight the importance of our protocol involving controlled dynamic balance perturbations, as increasing the task’s challenge allowed us to distinguish differences in motor patterns between the analyzed groups.

Overall, the findings of the present work align with broader evidence linking sarcopenia to reduced type II fiber presence and impaired force generation [4, 43–45]. The difference in MDF slope in the TA muscle may reflect altered fatigue dynamics or compensatory mechanisms in distal musculature. A decline in frequency is a well-established marker of muscle fatigue [32, 46, 47]. In this study, only the control group showed this pattern, while the sarcopenic group did not. This difference may reflect the altered muscle fiber composition caused by sarcopenia, which preferentially depletes type II fibers—key for fast, forceful movements—while largely preserving type I fibers, which are more fatigue-resistant [4, 43–45, 48].

Notably, the consistently lower SAMPEN values in the pre/sarcopenic group—particularly in GL, GM, and SO—indicate reduced signal complexity, which may reflect less adaptable or more stereotyped neuromuscular control. This reduction in complexity has been previously observed in sarcopenic patients and linked to the muscle aging process [26, 49]. The SO muscle, showing significant differences in both value and slope, appears especially sensitive to early neuromuscular decline, highlighting its potential as a biomarker for detecting sarcopenic-related deterioration in postural control [50, 51].

The differences observed in the shape parameters of the sEMG signal distributions during the Post-stab epoch may reflect distinct underlying neuromuscular strategies between the pre/sarcopenic and control groups, particularly in terms of motor unit (MU) synchronization and recruitment. Prior work has shown that MU synchronization alters the probability density function (PDF) of sEMG signals, typically increasing signal non-Gaussianity and accentuating the tails of the distribution [33, 39]. In our study, the pre/sarcopenic group exhibited higher RSD and LSD values, indicating broader signal tails and a more Laplacian-like PDF shape, which has been associated with increased synchronization and decreased variability in MU activation patterns. Such a pattern could represent a compensatory response or a sign of neuromuscular deterioration, where fewer MUs are active, but fire more synchronously, leading to sharper peaks and troughs in the signal. Conversely, the higher CSD values observed in the control group suggest a more distributed and variable activation in the central portion of the PDF, potentially reflecting greater MU recruitment and more flexible modulation during the stabilization phase. These findings align with simulation-based studies using core shape modelling (CSM), which showed that increased MU synchronization during contraction modifies the PDF shape, especially in the tails [39]. Our results suggest that early sarcopenia may disrupt this balance, favoring more stereotyped and synchronized firing patterns that degrade the fine control needed for adaptive postural stabilization. The significant decrease in the LSD parameter observed in the GL muscle among pre/sarcopenic individuals aligns with findings from previous research. Specifically, the study by [52] reported a similar pattern, indicating that alterations in the tail regions of the sEMG signal’s probability density function (PDF) may be indicative of neuromuscular changes associated with aging, which is something inherently characteristic of sarcopenia [4].

The sEMG signal differences observed in our study, based on temporal, spectral, and core shape features, reflect underlying neuromuscular impairments that are functionally significant and clinically relevant. These signal characteristics, including delayed onset, reduced frequency content, and altered signal complexity, are indicative of impaired muscle recruitment strategies and diminished neuromuscular responsiveness, which are commonly associated with sarcopenia and aging-related motor decline [6]. Importantly, several studies have established a clear relationship between such EMG alterations and increased fall risk. For instance, a large prospective study by AB Gadelha et al. (2018) found that the incidence of falls increased progressively across sarcopenia stages: 15.4% in pre-sarcopenic, 40.7% in sarcopenic, and 72.0% in severely sarcopenic individuals over an 18-month follow-up period [53]. These findings underscore the clinical importance of early neuromuscular dysfunction, even in the absence of marked muscle mass loss.

These electrophysiological signatures are consistent with reduced motor unit recruitment and firing variability, both of which are critical for adaptive balance responses [54]. Previous studies have linked specific EMG patterns, such as prolonged activation latencies and diminished co-contraction efficiency, with impaired reactive postural control in older adults [55]. Our findings further corroborate this by demonstrating distinct sEMG signal profiles in sarcopenic individuals during dynamic stabilization, which may not be evident during static assessments. As a result, sEMG-based metrics obtained during controlled perturbation tasks may serve as sensitive biomarkers for identifying individuals at elevated fall risk, thereby augmenting traditional clinical evaluations and informing personalized fall-prevention strategies.

While our current study was not longitudinal and did not directly measure fall incidence, the temporal and spectral sEMG parameters are linked to impaired balance recovery and increased fall risk [56–58]. Future work could build on these results by integrating EMG features into multifactorial fall risk assessment models and testing their predictive validity in real-world settings.

The prominence of group differences in the Post-stab epoch may reflect the increased reliance on complex neuromuscular strategies required for sustained postural stabilization following the initial perturbation. Unlike earlier epochs, which are dominated by anticipatory (Pre-prep, Pre-vol) or reflexive (Auto-resp) mechanisms, the Post-stab phase (350–2350 ms post-perturbation) engages a prolonged period of adaptive, feedback-driven, and voluntary control to reestablish and maintain balance [59]. This phase demands a coordinated integration of sensorimotor information, dynamic muscle recruitment, and fine-tuned adjustments—capacities that might be compromised in individuals with early sarcopenia [60]. The consistent differences observed in signal amplitude, frequency content, entropy, and distributional shape during this epoch suggest that the neuromuscular system’s ability to adaptively stabilize posture over time is more affected by sarcopenic changes than the more automatic or anticipatory phases of the task [26]. Therefore, the Post-stab epoch may serve as a sensitive window to detect subtle functional declines in postural control associated with early neuromuscular deterioration. During the early phases of the task—particularly the Pre-prep and Pre-vol epochs—the body may engage in exaggerated or overcompensated anticipatory strategies to prepare for the perturbation, especially in individuals with impaired neuromuscular control who may attempt to “preemptively” counteract instability [20, 34]. These anticipatory efforts might transiently mask underlying deficits by boosting muscle activation or co-contraction. However, once the perturbation occurs and the system transitions into the stabilization phase, this compensatory effort may recede, revealing diminished adaptability and reduced neuromuscular efficiency in the pre-sarcopenic group. The Post-stab epoch, being a prolonged window involving both voluntary and feedback-driven control, likely captures this unmasking of underlying deficits, making it particularly sensitive for detecting group differences in motor control performance.

The greater number of significant findings in the Post-stab phase is unlikely to be solely due to its longer duration. The Pre-prep phase, of equal length, did not show similar results, and combining shorter epochs into a longer one did not enhance discriminative power. Our segmentation reflects distinct postural control phases, and the selected features are robust to signal length or were normalized. Therefore, the findings likely reflect genuine physiological differences rather than duration effects.

We believe that we obtained these results due to the strict control of the balance perturbation protocol when we instructed all participants to maintain the same initial foot position after the perturbation. Considering the strategies for maintaining posture, keeping the feet in the same position is more challenging than taking a step, and perhaps the pre/sarcopenic individuals might have already adapted their postural strategy by opting to take a step to recover balance in their daily activities. However, this possibility was not assessed in our study.

Despite the promising findings, several limitations of this study should be acknowledged. Previous research has consistently demonstrated a higher prevalence of sarcopenia among women compared to men, particularly in older populations, which may be attributed to sex-related differences in muscle mass, hormonal decline, and physical activity levels [61, 62]. Thus, the predominance of women with sarcopenia in our study population is representative of this broader epidemiological trend. Moreover, no significant differences were observed between men and women in the sEMG parameters during the Post-stab epoch, which was the primary focus of our analysis. However, we acknowledge that a more balanced sex distribution would enhance the generalizability of our findings. Future studies should prioritize larger and more diverse cohorts to enable stratified analyses by sex and sarcopenia severity, which may reveal distinct neuromuscular adaptation profiles relevant for personalized clinical interventions. Expanding the population and incorporating longitudinal designs will be essential to validate these biomarkers and explore their predictive value for functional decline or responsiveness to intervention. In addition, future research should consider validating the electrophysiological features derived from sEMG against direct measures of muscle composition, such as magnetic resonance imaging or muscle biopsy, to better ground these biomarkers in underlying structural and histological changes. These steps will strengthen the clinical relevance of sEMG-based assessments and support their potential integration into early screening and personalized management strategies for sarcopenia.

## Conclusions

This study aimed to investigate neuromuscular adaptations in individuals with pre-sarcopenia and sarcopenia during postural balance tasks, using sEMG signal features as potential biomarkers of early motor decline. Our findings highlight significant differences between control and pre/sarcopenic groups, especially during the Post-stab epoch, a phase dominated by feedback-driven and voluntary stabilization. Lower RMS values and reduced signal complexity (SAMPEN) in the pre/sarcopenic group suggest diminished muscle activation and less adaptable motor control. Moreover, alterations in the shape of the sEMG signal’s probability density function, including higher tail distances (RSD and LSD) and lower central shape distances (CSD), point toward increased motor unit synchronization and reduced recruitment variability—hallmarks of early neuromuscular deterioration.

The Post-stab epoch emerged as the most sensitive window for detecting these changes, likely due to the reduced influence of anticipatory compensatory strategies and the increased demand for sustained neuromuscular coordination. These results reinforce the potential of sEMG-derived features, including PDF shape metrics, as non-invasive, functional biomarkers for identifying subtle impairments in motor control associated with aging and sarcopenia. Future work should focus on validating these findings in larger and more diverse populations and exploring their clinical application in early diagnosis and intervention monitoring.

## Data Availability

The data that support the findings of this study are available from the corresponding author upon reasonable request.

## Appendix

**Table 6:** Spearman correlation coefficients between sEMG parameters and cofactors age and BMI, across five epochs (Pre-prep, Pre-vol, Auto-resp, Stab, and Post-stab) for each muscle (SO, GM, GL and TA). Statistically significant correlations (*p* < 0.05) are indicated in bold with (*)

		**Pre-prep**		**Pre-vol**		**Auto-resp**		**Stab**		**Post-stab**	
		**BMI**	**Age**	**BMI**	**Age**	**BMI**	**Age**	**BMI**	**Age**	**BMI**	**Age**
**RMS**	**SO**	0.17	0.15	0.14	0.18	− 0.06	0.16	− 0.14	0.13	− 0.21	0.05
	**GM**	− 0.16	0.29	− 0.13	0.37	− 0.28	0.09	− 0.33	0.25	− 0.21	− 0.09
	**GL**	0.17	0.21	0.05	0.31	0.15	0.13	− 0.23	0.18	− 0.10	0.03
	**TA**	− 0.19	− 0.19	− 0.01	− 0.20	− 0.06	− 0.08	0.05	0.09	0.06	0.32
**MDF**	**SO**	− 0.25	**0.43***	− 0.24	0.37	− 0.13	0.23	0.06	0.37	− 0.18	0.27
	**GM**	0.14	0.05	0.07	− 0.06	− 0.03	0.03	0.14	− 0.06	0.16	0.00
	**GL**	− 0.07	0.27	0.14	0.01	− 0.02	0.01	0.12	0.05	0.27	0.14
	**TA**	0.09	0.07	0.14	− 0.06	0.34	0.04	0.15	0.06	0.05	− 0.05
**SAMPEN**	**SO**	− 0.30	0.17	− 0.32	**0.40***	− 0.06	0.27	0.31	0.09	0.14	− 0.11
	**GM**	0.20	− 0.19	0.14	− 0.25	0.12	0.33	0.25	0.19	0.27	− 0.05
	**GL**	− 0.05	− 0.08	0.16	− 0.28	− 0.05	0.15	**0.43***	0.05	0.20	− 0.04
	**TA**	0.00	− 0.06	0.05	− 0.12	0.31	− 0.07	0.05	− 0.07	− 0.08	− 0.35
**CSD**	**SO**	− 0.16	0.02	− 0.03	0.02	− 0.10	− 0.19	− 0.20	− 0.12	− 0.28	0.09
	**GM**	− 0.06	0.10	− 0.30	− 0.02	− 0.23	** − 0.49***	** − 0.44***	− 0.29	− 0.21	− 0.18
	**GL**	− 0.07	− 0.06	− 0.25	0.13	0.18	− 0.14	− 0.01	0.25	0.10	− 0.05
	**TA**	− 0.04	0.14	− 0.17	0.08	− 0.01	0.23	− 0.13	0.02	0.03	0.20
**RSD**	**SO**	0.09	0.09	0.07	− 0.11	− 0.05	− 0.14	− 0.05	− 0.09	− 0.11	0.26
	**GM**	− 0.24	0.01	0.01	0.14	− 0.21	− 0.14	− 0.18	− 0.03	− 0.18	0.16
	**GL**	0.19	0.12	0.12	0.11	0.04	− 0.13	− 0.15	− 0.04	0.21	0.08
	**TA**	0.14	0.11	0.04	0.10	0.15	0.03	0.06	0.00	0.24	0.20
**LSD**	**SO**	− 0.03	0.13	0.03	− 0.02	0.21	− 0.18	− 0.20	0.05	− 0.11	0.01
	**GM**	0.15	− 0.12	0.21	0.02	0.08	− 0.26	0.05	− 0.24	− 0.03	0.05
	**GL**	0.06	0.02	− 0.13	− 0.16	− 0.20	− 0.04	− 0.06	0.05	− 0.16	− 0.04
	**TA**	0.06	0.14	− 0.04	0.07	− 0.17	0.25	− 0.11	0.23	− 0.01	0.33

**Table 7:** *P*-values from the statistical analysis comparing men vs. women across sEMG parameters, muscles, and epochs. Statistically significant *p*-values (*p* < 0.05) in bold

Muscle	Epoch	RMS	MDF	SAMPEN	CSD	RSD	LSD
**TA**	**Pre-prep**	**0.025**	0.303	**0.025**	0.052	**0.029**	0.719
	**Pre-vol**	**0.006**	0.633	**0.003**	**0.047**	**0.009**	0.649
	**Auto-resp**	0.075	0.940	0.152	0.065	**0.007**	0.905
	**Stab**	0.328	0.670	0.075	0.157	0.119	0.867
	**Post-stab**	0.821	0.633	0.498	0.350	0.077	0.684
**GL**	**Pre-prep**	0.247	0.269	0.662	0.792	0.755	0.401
	**Pre-vol**	0.425	0.625	0.898	0.517	0.829	1.000
	**Auto-resp**	0.269	0.292	0.554	0.172	0.943	0.792
	**Stab**	0.662	0.341	0.554	0.172	0.649	0.375
	**Post-stab**	0.173	0.939	0.554	0.281	1.000	0.221
**GM**	**Pre-prep**	0.807	0.683	0.807	0.581	0.684	0.401
	**Pre-vol**	0.531	0.115	0.807	0.052	0.303	0.098
	**Auto-resp**	0.644	0.261	0.765	0.981	0.615	1.000
	**Stab**	0.935	0.338	0.397	0.755	0.457	0.204
	**Post-stab**	0.129	0.605	0.935	0.517	0.755	0.429
**SO**	**Pre-prep**	0.098	0.867	0.072	0.615	0.089	0.429
	**Pre-vol**	0.157	0.457	**0.026**	0.684	0.119	0.905
	**Auto-resp**	0.058	0.719	0.119	0.649	0.108	0.649
	**Stab**	0.829	0.684	0.755	0.303	0.240	0.072
	**Post-stab**	0.187	0.649	0.829	0.867	0.221	0.072
